# Survival of Mexican Children with Acute Myeloid Leukaemia Who Received Early Intensification Chemotherapy and an Autologous Transplant

**DOI:** 10.1155/2015/940278

**Published:** 2015-03-02

**Authors:** Elva Jiménez-Hernández, María Teresa Dueñas-González, José Arellano-Galindo, María Elena Medrano-Ortíz-De-Zárate, Vilma Carolina Bekker-Méndez, Adolfina Berges-García, Karina Solís-Labastida, Berenice Sánchez-Jara, Héctor Manuel Tiznado-García, Ethel Zulie Jaimes-Reyes, Xochiketzalli García-Jiménez, Laura Espinoza-Hernández, Nora Nancy Núñez-Villegas, Sergio Franco-Ornelas, Ruy Xavier Pérez-Casillas, Octavio Martínez Villegas, Teresa Marin Palomares, Juan Manuel Mejía-Aranguré

**Affiliations:** ^1^Departamento de Hematología Pediátrica, Unidad Médica de Alta Especialidad (UMAE), Centro Médico Nacional “La Raza”, Instituto Mexicano del Seguro Social (IMSS), Avenida Jacarandas Esquina Vallejo, S/N, Colonia La Raza, 02990 México, DF, Mexico; ^2^Laboratorio de Investigación, Hospital Infantil de México “Federico Gómez”, Secretaría de Salud, Calle Doctor Marquez 162, Colonia Doctores, Delegación Cuauhtémoc, 06720 México, DF, Mexico; ^3^Hospital de Oncología, Centro Médico Nacional “Siglo XXI”, IMSS, Avenida Cuauhtemoc 330, 06720 México, DF, Mexico; ^4^Unidad de Investigación en Inmunología, Unidad Médica de Alta Especialidad (UMAE), Centro Médico Nacional “La Raza”, Instituto Mexicano del Seguro Social (IMSS), Avenida Jacarandas Esquina Vallejo, S/N, Colonia La Raza, 02990 México, DF, Mexico; ^5^Hematología Pediátrica, Centro Médico Nacional “Siglo XXI”, IMSS, Avenida Cuauhtemoc 330, 06720 México, DF, Mexico; ^6^Facultad de Medicina, Universidad Nacional Autónoma de México, Avenida Universidad 3000, 04510 México, DF, Mexico; ^7^Unidad de Investigación en Epidemiología Clínica, UMAE Hospital de Pediatría, Centro Médico Nacional “Siglo XXI”, IMSS, Avenida Cuauhtemoc 330, 4to Piso Edificio de la Academia Nacional de Medicina, 06720 México, DF, Mexico

## Abstract

*Background*. In Mexico and other developing countries, few reports of the survival of children with acute leukaemia exist. *Objective*. We aimed at comparing the disease-free survival of children with acute myeloid leukaemia who, in addition to being treated with the Latin American protocol of chemotherapy and an autologous transplant, either underwent early intensified chemotherapy or did not undergo such treatment. *Procedure*. This was a cohort study with a historical control group, forty patients, less than 16 years old. Group A (20 patients), diagnosed in the period 2005–2007, was treated with the Latin American protocol of chemotherapy with an autologous transplant plus early intensified chemotherapy: high doses of cytarabine and mitoxantrone. Group B (20 patients), diagnosed in the period 1999–2004, was treated as Group A, but without the early intensified chemotherapy. *Results*. Relapse-free survival for Group A was 90% whereas that for Group B it was 60% (*P* = 0.041). Overall survival for Group A (18, 90%) was higher than that for Group B (60%). Complete remission continued for two years of follow-up. *Conclusions*. Relapse-free survival for paediatric patients treated with the Latin American protocol of chemotherapy with an autologous transplant plus early intensified chemotherapy was higher than that for those who did not receive early intensified chemotherapy.

## 1. Background

The importance of acute myeloid leukaemia (AML) is evident because, although comprising only 15%–20% of childhood leukaemias, the mortality rates of AML account for up to 30% of leukaemia-related deaths [1–3]. In the past three decades, the prognosis of paediatric AML has improved significantly because of progress in the treatment of this disease, principally through the introduction of regimens of multidrug use and of intensification therapy and through improvement in postremission therapy and in support care. Despite these improvements, the accumulated risk of relapse is still approximately 30%–40% [4].

When administered alone, chemotherapy in paediatric AML has improved the probability of event-free survival (EFS); however, even in the best series published in the last decade, EFS reached only 31%–54% with the probability of overall survival (OS) between 36% and 66% [5–17]. When chemotherapy is combined with autologous haematopoietic transplantation, similar results are obtained; however, the importance of this combinatory treatment lies in its demonstrated ability to lower the risk of relapse [18–20]. Although allogeneic haematopoietic transplantation offers a higher probability of cure (up to 67%), its use in primary remission in groups with good risk is not recommended, because of its morbidity-mortality and because its use has not shown better results than chemotherapy alone [8, 12, 16, 19, 21, 22]. When the patients are stratified by independent prognostic indicators, groups with chromosomal aberrations indicative of good risk, such as t(8:21), inv (16), and t(15:17), can reach an OS of 91%, 92%, and 87%, respectively [23, 24].

Although the results with autologous transplants are similar to those with chemotherapy alone and their use is controversial, some groups that have obtained results either superior to those of chemotherapy alone or similar to those of allogeneic transplant retain their interest in the use of autologous transplants [25–30], because not only is the need for an HLA-identical donor obviated, but also the potentially fatal graft versus host disease is avoided.

However, there are disadvantages, such as a lack of effect of the graft against the leukaemia and the risk of reinfusion of leukaemia cells, bringing with them a greater risk of relapse. To reduce the latter possibility, various techniques of purge* ex vivo* or purge* in vivo* have been developed [31–34]. With the purge* in vivo*, together with the harvest of peripheral blood stem cells (PBSC), the risk of harvesting neoplastic cells is reduced; as a consequence, the risk of relapse from the disease is also reduced [34, 35]. When a patient previously received various cycles of intensification chemotherapy, even better results have been obtained [25, 27, 36, 37].

In the present study, the patients with AML, starting in 1999, received chemotherapy (called the Latin American protocol) that was based on the protocol of the German group (AML-Berlin-Frankfurt-Münster (BFM) 87 [38]) plus autologous transplantation in first remission, resulting in a five-year survival rate of 60%; starting in 2005, patients received, in addition to this standard treatment, a cycle of early intensification (EI) chemotherapy with high doses of cytarabine (Ara-C) and mitoxantrone (HAM) (AML-BFM 93) [39]. The objective of the present study was to compare the disease-free survival of children with AML who, in addition to being treated with the Latin American protocol of chemotherapy and an autologous transplant, either underwent EI chemotherapy or did not undergo such treatment.

## 2. Methods

### 2.1. Study Design

Cohort study for assessing survival: the Hematological Service of the General Hospital of the National Medical Center “La Raza” in Mexico City is the facility that treats the greatest number of patients with leukaemia in the whole Mexico. This project was approved by the Ethics and Research Committee of the General Hospital of the National Medical Center “La Raza,” Mexico City (number R2008-3502-63). Written consent from the parents of the patients was obtained when the children were first admitted to the hospital.

### 2.2. Patient Groups

**Figure 1:**
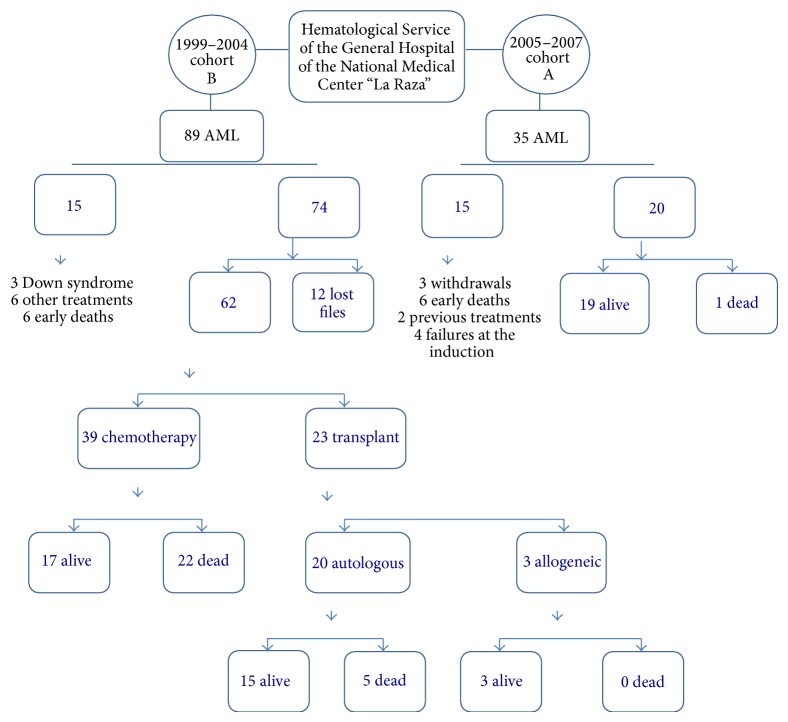
Patient selection. Group A (20 patients), diagnosed in the period 2005–2007, was treated with the Latin American protocol of chemotherapy with an autologous transplant plus early intensified chemotherapy: high doses of cytarabine and mitoxantrone (HAM). Group B (20 patients), diagnosed in the period 1999–2004, was treated as Group A but without the early intensified chemotherapy. AML: acute myeloid leukaemia.

All patients were less than 16-year-old. Group A (*n* = 20) comprised those patients who had been diagnosed with AML for the first time from January 2005 to December 2007. Group B (*n* = 20) consisted of those patients diagnosed with AML for the first time from January 1999 to December 2004. The composition of the cohorts is described in Figure 1. The patients were included when they the complete remission in all subtypes since M0 to M7 was reached and only to M3 when the patients had the second remission reached or when the* PLM/RARα* was negative.

The French-American-British (FAB) classification was used for the initial diagnosis and the microscopy determinations were corroborated by at least three haematologist observers; the subtypes, M0 and M7, were confirmed by immunologic methods; bone marrow aspirates taken on Day 15 were examined by two or more haematologists.

### 2.3. Risk Classification

The classification of risk was conducted according to the criteria of group AML-BFM 93 [40]. Patients at low risk were those with morphological subtypes M1, M2 (with Auer bodies) or t(8:21), as well as M4 with eosinophilia and 16 inversion in the karyotype, and if they had <5% blast cells in the bone marrow on Day 15 of induction of remission (IR). Patients with high risk were those, that morphologically were clasified as M1, M2, or M4, additionally in patients M4 and without characteristic mentioned above. With either >5% blast cells in the bone marrow on Day 15 of IR; with monosomy 7, 5 in the karyotype; or with complex karyotypes.

### 2.4. Response Criteria

The criteria used were those of the International Working Group for the Diagnosis and Treatment of AML [41].* Complete haematological remission*: no clinical evidence of leukaemia; bone marrow with restoration of normal haematopoiesis, with <5% blast cells; and, in the peripheral blood, ≥1 500 neutrophils/*μ*L and ≥100 000 platelets/*μ*L without transfusion.* Early death*: if death occurred when treatment was not initiated, or if death occurred less than seven days after having finished the first cycle of IR.* Death during treatment*: death during treatment with hypoplastic bone marrow.* Therapeutic failure*: persistence of leukaemia after having received two cycles of chemotherapy for IR.* Relapse-free survival (DFS)*: the time that elapsed from complete remission until the haematological, genetic, or molecular detection of relapse of the disease.* Relapse*: after remission was declared, reemergence of the disease as indicated by blast cells in the peripheral blood, ≥5% of blast cells in the bone marrow, or extramedullary infiltration.* Overall survival (OS)*: the time elapsed from the date of diagnosis until the most recent follow-up. Toxicity of the chemotherapy was evaluated in accordance with the criteria of the US National Cancer Institute (NCI) [42].

### 2.5. Treatment

The schematic of chemotherapy (Supplemental Figure I, see Supplementary Material available online at http://dx.doi.org/10.1155/2015/940278) which for Group B was based on the Latin American protocol (AML-BFM 87) [38] and which for Group A was based on AML-BFM 93 [39], with the following modifications: 6-thioguanine was not used in the consolidation, because this drug is not available in Mexico; prophylactic radiation of the central nervous system (CNS) was not administered, because it was not considered necessary as, historically, the number of relapses in the Hematological Service is very low for the type of patients treated. Patients in both groups underwent an autologous transplant in first complete remission (CR1). The toxicity related to this regimen was investigated by using the NCI criteria [42].

### 2.6. Statistical Analysis

For the quantitative variables without normal distribution, a Mann-Whitney *U* test was used for comparison of two independent groups; for qualitative variables, *χ* ^2^ or Fisher's exact test was used. *P* < 0.05 was considered statistically significant. The Kaplan-Meier method was used to construct survival plots; a log-rank test was used for comparison between groups.

We analysed the following potential variables that influence relapse: age, sex, leukocyte count at diagnosis, response on Day 15 of the IR, time from diagnosis to complete remission (CR), low or high risk, the cycles of chemotherapy required to achieve remission, time of remission to transplant, quantity of MNC × 10^8^/kg, and quantity of CD34+ × 10^6^/kg. For these variables, the crude relative risk (CRR), the CRR by strata, and the CRR, adjusted by Mantel-Haenszel statistics, with confidence intervals of 95%, were calculated in two-by-two tables. The statistical package SPSS version 21 was used.

## 3. Results

### 3.1. General Characteristics of the Patients

In this study, no significant differences were found between the groups (20 patients per group) for any of the general or clinical characteristics analysed (Table 1). For the two groups, the median age was nine years; the distribution by sex was similar; the median leukocyte count at diagnosis was 28 250/*μ*L for Group A and 26 900/*μ*L for Group B (*P* = 0.698). In both groups, the most common morphological subtypes were M4 and M5, which together comprised 55% of the cases in Group A and 60% in Group B. Of the 40 patients, 35% had a normal karyotype; five had a low-risk karyotype (t(8:21)), and three had a high-risk karyotype. According to the risk classification of the BFM group, 34 patients had high-risk parameters.

### 3.2. Primary Results: Response to Treatment

#### 3.2.1. Relapse-Free Survival after an Autologous Transplant

**Figure 2:**
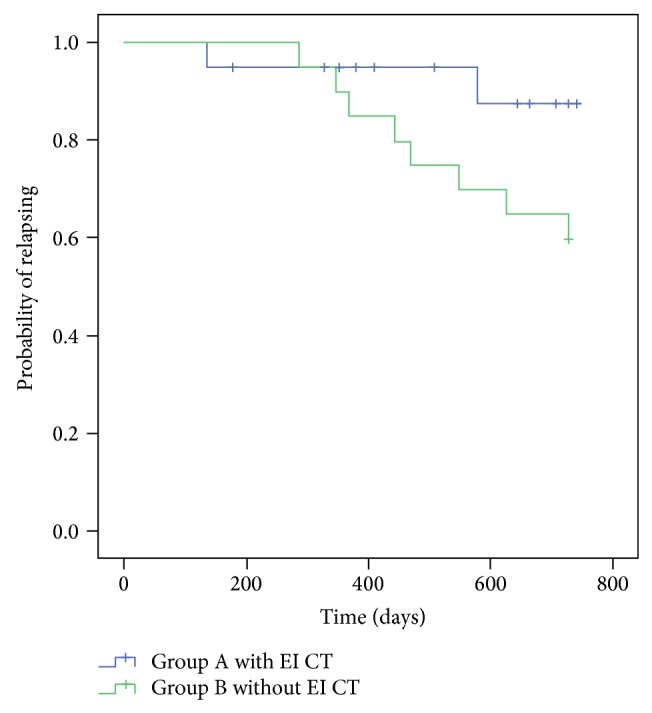
Relapse-free survival after autologous transplant for patients treated with early intensification chemotherapy or not. EI: early intensification; CT: chemotherapy. *P* = 0.09.

Relapse-free survival (Figure 2) was 90% for Group A and 60% for Group B (*P* = 0.041); these patients were in complete continuous remission (CCR), with a median follow-up time of 405 days for Group A and 527 days for Group B. Of the 10 patients who suffered a relapse (Table 2), the relapse occurred in the bone marrow of eight, in the CNS of one, and in both sites in another; none of these 10 attained a second remission and all died from leukaemic activity.

#### 3.2.2. Overall Survival

**Figure 3:**
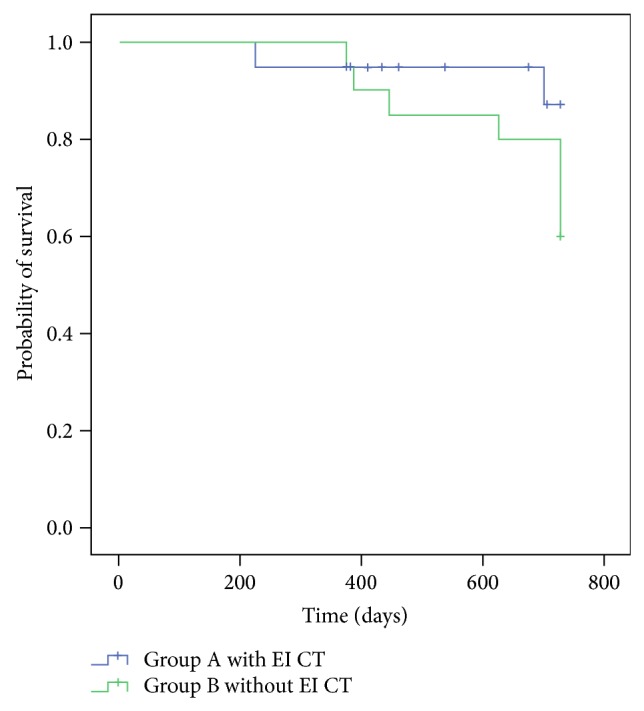
Overall survival at two years of follow-up for patients treated with early intensification chemotherapy or not. EI: early intensification; CT: chemotherapy. *P* = 0.13.

The overall survival (OS) (Figure 3) was 18 (90%) for Group A with a median duration of follow-up of 610 days (minimum, 226; maximum, 730). For Group B, the OS was 12 (60%), with a median follow-up of 675 days (minimum, 376; maximum, 730) (*P* = NS).

#### 3.2.3. Variables Associated with Relapse

Analysis of the variables that could influence relapse showed that none of these variables affected the risk, whether calculated as CRR or as that adjusted by Mantel-Haenszel statistics (Table 3).

### 3.3. Secondary Results: Toxicity of EI Chemotherapy in Group A

No patient died from treatment toxicity; nor were any excluded from the study for this reason. All 20 (100%) displayed fever and neutropenia and required empirical treatment with wide-spectrum antibiotics; 12 of these patients were given amphotericin B for having persistent fever for more than five days, despite the use of the antibiotics. Two other patients suffered pneumonia; two had neutropenic enterocolitis that responded to antibiotics; and only one developed systematic candidiasis, which was resolved with amphotericin B and that did not influence the transplant. All patients presented haemorrhage of the skin and mucosa; seven had blood in the digestive tract (haematemesis or bleeding from the rectum) that was resolved with the transfusion of platelets.

### 3.4. Harvest of Haematopoietic Stem Cells

The characteristics of the harvested of autologous hematopoietic stem cells from pediatric patients are shown in Supplemental Table I. Neither the MNC nor the CD34+ cell count differed between groups (*P* = 0.70). There were no differences between groups for any of the other parameters studied.

### 3.5. Transplant of Haematopoietic Stem Cells

Of the 20 patients in Group A, who were programmed for an autologous transplant, a patient with AML-M2 (karyotype t(9;11)p22;q23) did not have the procedure because of a bone marrow relapse; the patient died as a result of infection and leukaemic activity eight months after diagnosis. It is for this reason that only 19 patients in Group A received transplants. (No patient died from the toxicity of the EI. One other patient died from infection at another stage of treatment because of leukaemic activity.) For these 19 patients, the median time from complete remission to transplant was 9.9 months (minimum, 6.1; maximum, 12.8), whereas for Group B (*n* = 20), the median was 8.6 months (minimum, 5.6; maximum 15.7) (*P* = 0.235); thus, EI chemotherapy did not cause a major delay in transplantation.

All these patients received an adequate transplant; the median number of days needed to reach a polymorphonuclear cell count ≥0.5 × 10^9^/L was 18 days (minimum, 12; maximum, 35) for Group A and 18 days (minimum, 14; maximum, 38) for Group B (*P* = 0.749); to reach a platelet cell count ≥50 × 10^9^/L, without transfusion, the median number of days needed was 23 (minimum, 18; maximum, 54). The patients received G-CSF after the infusion of the harvested cells; on Day +18, five patients from Group A and four from Group B did not have a neutrophil count ≥0.5 × 10^9^/L.

### 3.6. Complications and Mortality Related to the Transplant

Of the 39 patients who received transplants, 12 (31%) did not present any complications; 12 (31%) developed a fever that lasted more than seven days and with neither a localized site of infection nor an isolated microorganism, thus warranting the use of wide-spectrum antibiotics (ceftazidime/amikacin or vancomycin); and eight (21%) required amphotericin B. Mortality as a result of transplantation was 0%.

## 4. Discussion

The study AML-BFM 93 [39] found that the combination of idarubicin in the induction and the intensification therapy with HAM reduces the risk of relapse in patients at high risk. The other factor that probably was potentiated by the EI was the prolonged average time (270 days) from remission to transplant, as was demonstrated by Locatelli et al. [43]. In that multivariate analysis, it was shown that, of the children who had transplants at ≥170 days after the first CR, 60% ± 3% had a leukaemia-free survival during five years of follow-up. This beneficial effect may be the result of two principal factors: (1) additional useful courses of consolidation to reduce the tumour burden and (2) the low risk of mortality related to the transplants in these patients, which is because of the sufficient time for organic recovery before the transplantation.

Other studies have demonstrated the effectiveness of HAM in treating AML, both in children and in adults. The GALGB study was the first to show the effect of cytarabine at a standard dose (100 mg/m^2^), intermediate dose (400 mg/m^2^), or high dose (3 g/m^2^) as treatment postremission [44]. Thereafter, Arlin et al. [45] reported a higher average of CR after only one course of induction with Ara-C and mitoxantrone (3 × 12 mg/m^2^), when these drugs were administered after a standard regimen of daunorubicin (3 × 45 mg/m^2^) in adult patients with newly diagnosed AML. Büchner et al. [46] demonstrated that HAM as a course for second induction in adults benefits patients at high risk; here, we had similar results for high-risk paediatric patients. In the AML-BFM 93 study, Creutzig et al. [39] evaluated HAM as a first or second cycle of postinduction treatment, with the intention of improving the results in children with AML of high risk. They found no statistically significant difference between the results obtained with the early or late administration of HAM; nevertheless, upon comparison with results of the historic group (AML-BFM 87 [38]), the differences were greater in OS and RFS of up to five years (60% ± 3%, 51% ± 2%, and 62% ± 3%, resp.). Similar values were found in the present study.

In the present study, no patient died or was excluded because of toxicity of the treatment; nor was there a significant delay in conducting the autologous transplantation. These results differ from those of AML-BFM 93 [39]. Nonetheless, the incidence of deaths related to the therapy was similar to that in AML-BFM 87 [38]. Therefore, because of the efficacy of HAM and its tolerable average toxicity in children at high risk, in the study AML-BFM 98 [47], HAM was introduced as a second course of therapy for all paediatric patients with AML, including patients with standard risk, with the objective of improving the average survival.

An important finding emerged from the present study, namely, the positive role of EI therapy, followed by a course of consolidation and two courses of late intensification before the harvesting of PBSC and autologous transplant, resulting in a significantly lower estimated probability of relapse (10%) compared with that of patients in the control group that received PBSCT without EI (40%; *P* = 0.04) (relative risk = 0.25, 95% CI 0.06–1.03).

Intensive chemotherapy coupled with an autologous transplant in primary remission constitutes a good option for treatment. However, the results in the literature vary and are difficult to compare because of the heterogeneity of the studies. They differ in design, number of patients, age of patients, intensity of the courses of chemotherapy before of the transplant, accumulated doses of the most important drugs (anthracyclines, cytarabine, and etoposide), prophylaxis of the CNS, difference in the stratification of risk, the time at which the transplant was performed, the conditioning regimens, the origin of the stem cells, and the use (or not) of a purge [25–37]. In the present study, with the use of EI chemotherapy, a cycle of consolidation, and two late intensifications before the harvest and autologous transplant, the RFS at two years was 90% compared with 60% for the control group that had not received such treatment (*P* = 0.03; Table 2). Previously, the efficacy of EI as a purge* in vivo* in children and adults who were newly diagnosed with AML and who received PBSCT was evaluated [36]. They reported that, for the patients who received EI before the harvesting and the transplant compared with patients who did not, the DFS rates were 68.8% ± 10.27% versus 35.5% ± 12.6%, respectively (*P* = 0.04). They concluded that, with the use of EI and PBSCT, the risk of relapse was reduced significantly. However, the majority of research groups do not recommend an autologous transplant as postremission therapy in paediatric patients with AML in CR1, because they have not found any benefit compared with that of chemotherapy alone [18, 39, 47].

With the best support care, the mortality related to transplant toxicity has been reduced to <5%; in the present study, it was 0%, equal to that reported in the literature [25, 26, 29]. With the use of nonpurged and noncryopreserved PBSC in this study, the toxicity and the cost were reduced without interfering with the time of implant, as has been reported by Ruiz-Argüelles et al. [48] and Gómez-Almaguer [49]. Allogeneic transplant continues to be recommended for those paediatric patients with AML at high risk in CR1, who can count on having an HLA-identical family member, because the survival is 61% at five years of follow-up [50].

The following limitations apply to this study. Because the zero point for the cohort was defined when CR was achieved, it may be that those patients who were not included for reasons such as early death, failure of the IR, and nonacceptance of treatment were probably those at higher risk of relapse. Although the two groups had similar general and clinical characteristics, the comparison with the historic group may have been biased because of improvements over time, in support care, and with greater experience in management. In Group A, the follow-up time was short: not all the patients completed the minimum time established for follow-up (two years), which is the time of greatest risk of relapse; after then the probability of cure increases up to 80%. This was observed in the historic group of this study and was recently reported by Majhail et al. [20]. Finally, it is important to emphasize that there are various groups (e.g., in Argentina (GATLA), Chile (PINDA), and Mexico [17]) that do not include EI (HAM) in their chemotherapy plan. From the results of the present work, we recommend the use of EI (HAM) in chemotherapy.

## Supplementary Material

In the figure I, the schematic of chemotherapy is shown. That for Group B was based on the Latin American protocol (AML-BFM 87) [38] and that for Group A on AML-BFM 93. The treatment schedule was described with detail and the way as autologous transplant was done. In the table I the harvest of autologous haematopoietic stem cells is mentioned.

## Figures and Tables

**Table 1:** General and clinical characteristics of the paediatric patients with acute myeloid leukaemia.

Characteristic	Patients		*P*
	Group A with EI^a^	Group B without EI	
	(*n* = 20)	(*n* = 20)	
Age (years)			0.718
Median (min–max)	9 (2–15)	9.5 (2–15)	
Sex			0.525
Male	11 (55%)	11 (55%)	
Female	9 (45%)	9 (45%)	
Leukocyte count (10^3^/*μ*L)			0.698
Median	28 250	26 900	
Minimum–maximum	9 700–125 000	5 800–154 700	
Morphological subtype (FAB^b^)			0.941
M0, M1, M2	6 (30%)	4 (20%)	
M3	1 (5%)	1 (5%)	
M4, M5	11 (55%)	12 (60%)	
M6	1 (5%)	1 (5%)	
M7	1 (5%)	2 (10%)	
Karyotype			0.900
Normal	8 (40%)	6 (30%)	
t(8:21)aml/atg8	2 (10%)	3 (15%)	
t(15:4)q+	0 (0%)	1 (5%)	
t(9:22)abr/bcl	1 (5%)	1 (5%)	
t(9:11)p22,q23	1 (5%)	1 (5%)	
No data	8 (40%)	8 (40%)	
Risk (by karyotype)			0.870
Normal	4 (17%)	3 (13%)	
Intermediate	8 (33%)	6 (25%)	
High	2 (8%)	1 (4%)	
Risk (BFM^c^)			0.669
Low	3 (15%)	3 (15%)	
High	17 (85%)	17 (85%)	

^a^EI: early intensification.

^b^FAB: French-American-British classification.

^c^BFM: Berlin-Frankfurt-Münster study [39].

**Table 2:** Outcomes for paediatric patients with acute myeloid leukaemia, treated with early intensification chemotherapy or not.

Parameter	Patients		*P*
	Group A with EI^a^	Group B without EI	
	(*n* = 20)	(*n* = 20)	
CCR^b^	18 (90%)	12 (60%)	0.028
Relapse			
Pretransplant	1 (5%)	0 (0%)	
Posttransplant	1 (5%)	8 (40%)	0.031
Site of relapse			
BM^c^	2 (10%)	6 (30%)	0.030
CNS^d^	0 (0%)	1 (5%)	
BM + CNS	0 (0%)	1 (5%)	
Current state			
Alive with CCR	18 (90%)	12 (60%)	0.028
Dead	2 (10%)	8 (40%)	0.031
Cause of death			
Leukaemic activity	2 (100%)	8 (100%)	0.031
Related to APBT^e^	0 (0%)	0 (0%)	

^a^EI: early intensification.

^b^CCR: continued complete remission.

^c^BM: bone marrow.

^d^CNS: central nervous system.

^e^APBT: autologous peripheral blood transplant.

**Table 3:** Parameters analyzed for relapse adjusted by different prognostic factors.

Variable	Patients						
	Group A with EI^a^ (*n* = 20)		Group B without EI (*n* = 20)		Crude relative risk		
	No relapse	Relapse	No relapse	Relapse			
	*n* (%)	*n* (%)	*n* (%)	*n* (%)	CRR × S^b^ (CI 95%)	RRAMH^c^ (CI 95%)	
Age (years)							
<10	10 (90.9)	1 (9.1)	7 (58.3)	5 (41.7)	0.22 (0.03–1.59)		
>10	8 (88.9)	1 (11.1)	5 (62.5)	3 (37.5)	0.30 (0.04–2.31)	0.25	(0.06–1.11)
Sex							
Female	8 (88.9)	1 (11.1)	9 (100)	0 (0.0)	2.00 (0.21–18.98)		
Male	10 (90.9)	1 (9.1)	3 (27.3)	8 (72.7)	0.22 (0.06–0.84)	0.40	(0.14–1.11)
Leukocytes							
<50 000/*μ*L	15 (100)	0 (0.0)	10 (62.5)	6 (37.5)	0.15 (0.20–1.10)		
>50 000/*μ*L	3 (60.0)	2 (40.0)	2 (50.0)	2 (50.0)	1.14 (0.40–3.18)	0.47	(0.18–1.18)
BM^d^ Day 15							
**<**5% blasts	11 (91.7)	1 (8.3)	10 (71.4)	4 (28.6)	0.29 (0.37–2.26)		
>5% blasts	7 (87.5)	1 (12.5)	2 (33.3)	4 (66.7)	0.18 (0.02–1.27)	0.23	(0.05–0.94)
Risk							
Low	3 (100)	0 (0.0)	3 (100)	0 (0.0)	1.00 (0.08–11.9)		
High	15 (88.2)	2 (11.8)	9 (52.9)	8 (47.1)	0.33 (0.10–1.04)	0.40	(0.14–1.10)
Time of CR^e^							
<8 weeks	14 (93.3)	1 (6.7)	9 (64.3)	5 (35.7)	0.18 (0.02–1.40)		
>8 weeks	4 (80.0)	1 (20.0)	3 (50.0)	3 (50.0)	0.40 (0.59–2.74)	0.26	(0.06–1.04)
CT^f^ for CR							
One cycle	11 (91.7)	1 (8.3)	10 (66.7)	5 (33.3)	0.25 (0.03–1.86)		
Two cycles	7 (87.5)	1 (12.5)	2 (40.0)	3 (60.0)	0.20 (0.02–1.49)	0.23	(0.05–0.95)
CR upon APBT^g,h^							
>10 months	9 (90.0)	1 (10.0)	4 (57.1)	3 (42.9)	0.37 (0.87–1.61)		
<10 months	9 (100)	0 (0.0)	8 (61.5)	5 (38.5)	0.15 (0.02–1.14)	0.26	(0.82–0.83)
MNC^h,i^ (10^8^/kg)							
<5	14 (93.3)	1 (6.7)	8 (66.7)	4 (33.3)	0.32 (0.07–1.44)		
>5	4 (100)	0 (0.0)	4 (50.0)	4 (50.0)	0.33 (0.10–1.06)	0.33	(0.10–1.06)
CD34+ cells^h^ (10^6^/kg)							
>3	3 (100)	0 (0.0)	3 (60.0)	2 (40.0)	0.46 (0.06–3.23)		
<3	15 (93.8)	1 (6.3)	9 (60.0)	6 (40.0)	0.26 (0.06–1.12)	0.32	(0.10–1.00)

^a^EI: early intensification.

^b^CRR × S: crude relative risk by strata.

^c^CR (MH): relative risk, adjusted (Mantel-Haenszel).

^d^BM: bone marrow.

^e^CR: complete remission.

^f^CT: chemotherapy.

^g^APBT: autologous peripheral blood transplant.

^h^Only 19 patients in Group A received transplant (see text for details).

^i^MNC: mononuclear cell.
