# Supplementary material for: Survival of Mexican Children with Acute Myeloid Leukaemia Who Received Early Intensification Chemotherapy and an Autologous Transplant
Source: Biomed Res Int. 2015 Mar 2;2015:940278. doi: 10.1155/2015/940278 (PMC4363903; doi:10.1155/2015/940278)
Supplement: Supplementary file 1 — In the figure I, the schematic of chemotherapy is shown. That for Group B was based on the Latin American protocol (AML-BFM 87) [38] and that for Group A on AML-BFM 93. The treatment schedule was described with detail and the way as autologous transplant was done. In the table I the harvest of autologous haematopoietic stem cells is mentioned. [file 940278.f1.docx]

**AIE**

**HAM**

**Consolidation**

**(6 weeks)**

**HAE**

**(2)**

**Autologous transplant**

**Maintenance**

**(1 year)**

**(CNS 18 Gy)**

**CR**

**IR**

**EI**

| **Intrathecal chemotherapy calculated for age:** | | | | |
| --- | --- | --- | --- | --- |
| **Drug** | **Age range (years)** | | | |
|  | **<1** | **1–2** | **2.1–3.0** | **>3** |
| **Ara-C (mg)** | **20** | **30** | **50** | **70** |
| **Dexamethasone (mg)** | **2** | **4** | **4** | **4** |

**Supplemental Figure I.** **Chemotherapy schematic.**

IR: induction of remission; AIE: Ara-C, idarubicin, etoposide; CR: complete remission; EI: early intensification; HAM: high doses of Ara-C and mitoxantrone; HAE: high doses of Ara-C and etoposide; CNS: central nervous system.

***Treatment***

All patients had the same treatment schedule, the only difference being that Group A received early intensification. In the induction, cytosine arabinoside or cytarabine (Ara-C), idarubicin, and etoposide (AIE) were administered at the following doses: Ara-C, 100 mg/m^2^ in continuous infusion on Days 1 and 2, followed by 30 min infusion every 12 h on Days 3–8; idarubicin, 12 mg/m^2^ per one-hour infusion every 24 h on Days 3–5; and etoposide, 150 mg/m^2^ per 1 h infusion every 24 h on Days 6–8. EI consisted of high doses of Ara-C and mitoxantrone (HAM): Ara-C, 3 g/m^2^ per 3 h infusion every 12 h on Days 1–3 and mitoxantrone, 10 mg/m^2^ in 1 h infusion on Days 4 and 5.

Consolidation consisted of 6 mercaptopurine (60 mg/m^2^, orally, on Days 1–43); prednisone (40 mg/m^2^, orally, on Days 1–28); vincristine (1.5 mg/m^2^ on Days 1, 8, 15, and 22); daunorubicin (30 mg/m^2^ on Days 1, 8, 15, and 22); Ara-C (75 mg/m^2^ on Days 3–6, 10–13, 17–20, 24–27, 31–34, and 38–41); Ara-C and dexamethasone, both given intrathecally, on Days 1, 15, 29, and 43 (the dose was dependent on age; see Supplemental Figure I); and cyclophosphamide (500 mg/m^2^ on Days 29 and 43). Late intensification consisted of high doses of Ara-C and etoposide (HAE): Ara-C, 3 mg/m^2^ every 12 h on Days 1–3 and etoposide, 125 mg/m^2^ in 1 h infusion on Days 2 and 5. The same schedule was repeated when the patients achieved a haemoglobin level higher than 10 g/dL, total neutrophils above 1000/μL, and platelets above 100 000/μL. Thereafter, the patients, none of whom had an HLA-compatible family member, underwent an autologous transplant.

***Autologous transplant***

*Mobilization of haematopoietic stem cells*

Starting on Day –11, granulocyte colony-stimulating factor (G-CSF) was administered at 12 mg/kg/day, divided into two subcutaneous doses every 12 h for five days, with premedication given 20 min before by oral dose of paracetamol (10 mg/kg). Haematological biometry was conducted daily to monitor the increase in leukocytes; administration of the G-CSF was suspended when the concentration of leukocytes was ≥70 000/μL, or if the patient suffered an anaphylactic reaction.

*Collection of haematopoietic stem cells*

Before implantation of a Mahurkar catheter in the subclavian vein, haematopoietic stem cells were harvested on Days –8 and –7 with an aphaeresis machine (Baxter CS 3000) at a velocity of 50 mL/min per three volumes of blood. At harvest, stem cell viability, mononuclear cell (MNC) count, and CD34+ cell count were determined, and a blood culture was made. The harvested stem cells were transported to the Central Blood Bank of the Instituto Mexicano del Seguro Social (IMSS) and stored refrigerated (4 °C; 7 days). The characteristics of the harvested stem cells are shown in Supplemental Table I

***Conditioning regimen***

All patients received busulfan at 4 mg/kg on Days –7 to –4, given orally in four doses per day (total 16 mg/kg). Towards the end of the second harvest, cyclophosphamide (60 mg/kg on Days –3 and –2) was administered, with phenytoin given as prophylaxis for the convulsive crisis induced by the busulfan, with rest on Day –1.

***Infusion***

The harvested cells were infused on Days 0 and 1 via a central venous catheter for 15–20 min.

**Supplemental Table I. Harvest of autologous haematopoietic stem cells from paediatric patients**

| **Parameter** | **Patients** | | | | | | **P** |
| --- | --- | --- | --- | --- | --- | --- | --- |
|  | **Group A with EI^a^** | | | **Group A without EI** | | |  |
|  | **Median** | **Minimum** | **Maximum** | **Median** | **Minimum** | **Maximum** |  |
| MNC^b^ (10^8^/kg) | 5 | 2 | 10 | 5 | 3 | 11 | 0.728 |
| CD34+ cells (10^6^/kg) | 3 | 1 | 4 | 2 | 1 | 8 | 0.444 |
| % viability at end harvest | 97 | 95 | 98 | 96 | 94 | 97 | 0.739 |
| % viability pre-transplant | 89 | 82 | 93 | 90 | 83 | 94 | 0.749 |
| Day of leukocyte transplant | 18 | 12 | 35 | 18 | 14 | 38 | 0.749 |
| Platelets (>50/10^9^/L) | 23 | 18 | 54 | 22 | 14 | 58 | 0.649 |

^a^EI: early intensification

^b^MNC: mononuclear cells
